# Introducing conjoint analysis method into delayed lotteries studies: its validity and time stability are higher than in adjusting

**DOI:** 10.3389/fpsyg.2015.00023

**Published:** 2015-01-28

**Authors:** Michał Białek, Łukasz Markiewicz, Przemysław Sawicki

**Affiliations:** Economic Psychology Department, Centre for Economic Psychology and Decision Sciences, Kozminski UniversityWarsaw, Poland

**Keywords:** delay discounting, probability discounting, conjoint, adjusting, methodology

## Abstract

The delayed lotteries are much more common in everyday life than are pure lotteries. Usually, we need to wait to find out the outcome of the risky decision (e.g., investing in a stock market, engaging in a relationship). However, most research has studied the time discounting and probability discounting in isolation using the methodologies designed specifically to track changes in one parameter. Most commonly used method is adjusting, but its reported validity and time stability in research on discounting are suboptimal. The goal of this study was to introduce the novel method for analyzing delayed lotteries—conjoint analysis—which hypothetically is more suitable for analyzing individual preferences in this area. A set of two studies compared the conjoint analysis with adjusting. The results suggest that individual parameters of discounting strength estimated with conjoint have higher predictive value (Study 1 and 2), and they are more stable over time (Study 2) compared to adjusting. We discuss these findings, despite the exploratory character of reported studies, by suggesting that future research on delayed lotteries should be cross-validated using both methods.

## Introduction

Humans' decisions usually require managing both risk and delay, but these components are typically studied separately (Green and Myerson, 1996; Myerson et al., 2003; Murphy et al., 2011). Some researchers (Johnson et al., 2007; Weber et al., 2007; Appelt et al., 2011) have even suggested that all decisions with delayed outcome also contain the risk factor, because people are uncertain whether they receive the promised reward or even that they will still be alive on that date. Also, most real-life risky decisions bring delayed results, e.g., investments, dating an interesting person, career planning. Despite the fact that intertemporal choices and deciding under risk are among the most important topics in behavioral sciences (being widely popularized by Prospect Theory; Kahneman and Tversky, 1979; Tversky and Kahneman, 1992), the methodology used is still not sufficiently precise (Frederick et al., 2002; Odum, 2011a), especially for tasks that include combined risk and delay (Keren and Roelofsma, 1995; Yi et al., 2006; Ida and Goto, 2009a,b; Weatherly et al., in press). Our aim was to validate, specifically in this specific area of research, one of the most prominent method for measuring the discounting (adjusting) and compare it with less popular but hypothetically more reliable and valid method (conjoint). Reported here studies have an exploratory character, but they provide a broad insight into this methodological problem.

The “*adjusting method*” has gained higher popularity among psychology researchers, especially those who focus on discounting (Du et al., 2002; Białaszek and Ostaszewski, 2012; Green and Myerson, 2013; Green et al., 2013). In adjusting one has to make a choice between two alternatives (e.g., smaller certain and bigger uncertain gain) out of which one becomes adjusted in the next set of choices, but the process of adjusting does not obviate methodological difficulties, which we will discuss in more detail in the next section of the article.

The conjoint analysis method is popular in the marketing and applied psychology fields. It is based on the natural, real-life-alike tasks. Participants have to select the most (and sometimes also the least) preferred option among several presented, e.g., what chance of receiving $100 do you prefer: (a) 0.1 probability in 3 months, (b) 0.3 in 6 months, or (c) 0.05 in 1 week. The delay and probability are called “attributes,” and specific probability or delay is an “attribute level.” The utility (called part-worth) of specific attribute level is obtained indirectly based on previous choice by conducting the regression analysis. Conjoint method, compared with adjusting, requires a participant to make far less choices to provide enough data for stating his or hers preferences, e.g., in order to assess nine delayed lotteries (three delays × three probabilities) one has to make 54 choices (nine tasks, six steps each) using adjusting and only about 15–25 using conjoint method.

Adjusting and conjoint methods were not designed specifically to measure the preferences in delayed lotteries; instead, they originated from other fields of research. We assessed the quality of the data provided by those methods by evaluating its predictive quality and time stability. In the next section, we describe, in more detailed manner, how the preferences are calculated using the data provided by conjoint and adjusting methods.

## Methods

### Methods of measuring the discounting strength

#### Adjusting

**Figure 1:**
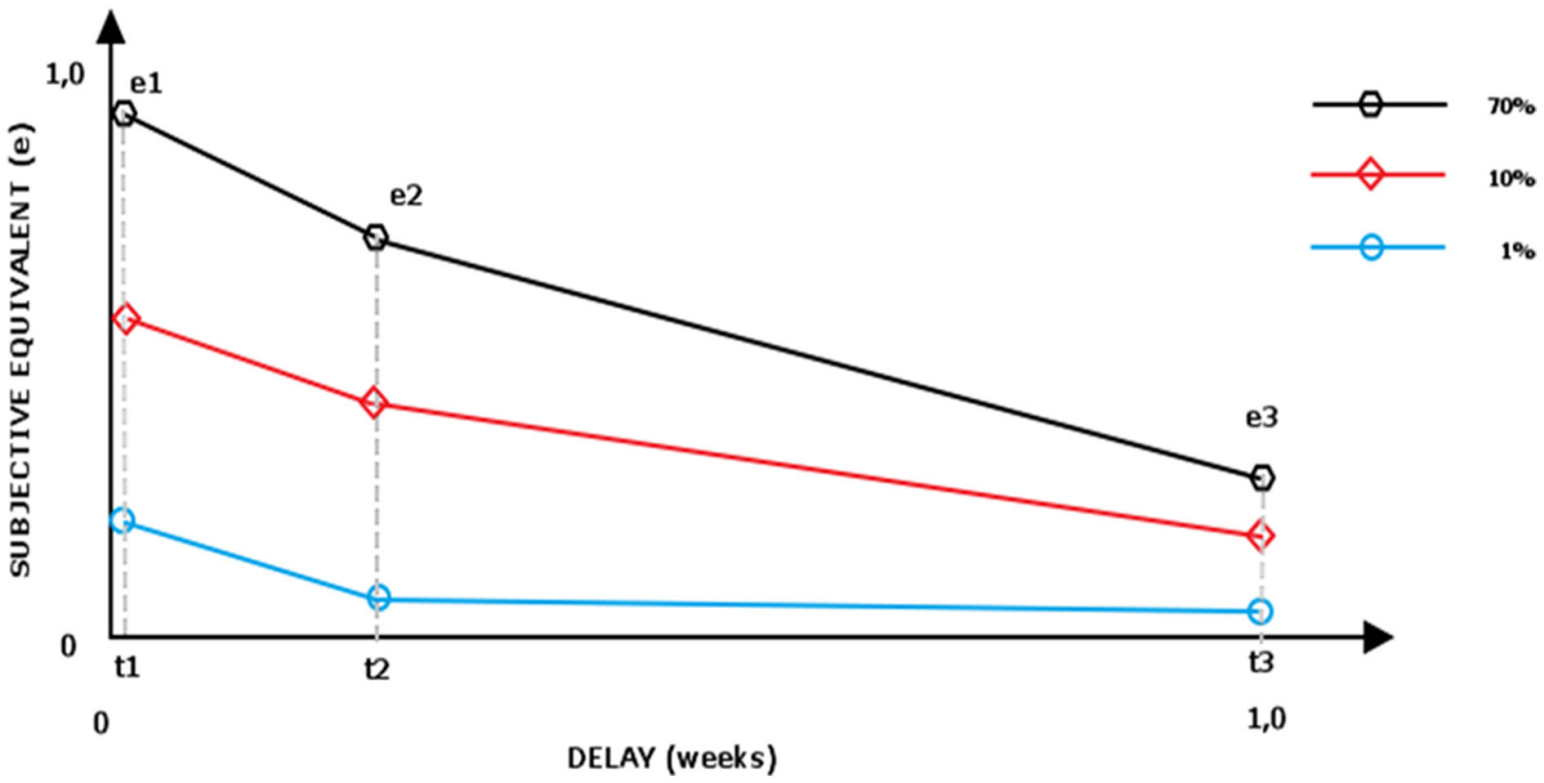
**Area under the curve for hypothetical three delayed lotteries (0.01;0.1;0.7)**.

In adjusting procedure the participants choose one alternative among two presented on the screen. Individuals choose one of two cards according to their preferences. The delayed and uncertain amount is constant, whereas the immediate and certain alternative change their value based on individuals' consecutive choices. If the participants choose the delayed lottery, the alternative increases (usually) by half of its previous value. To illustrate this procedure, let us follow an example: the presented set consists of two possible gains: (a) $5.000 in 6 months with 0.3 probability and (b) $2.500 now and certain. If the participant chose the immediate offer, it decreases by half of its previous value (to $1.250), but if he chose the delayed and uncertain alternative, the certain and immediate alternative rises (to increase its attractiveness) to $3.750. After making predefined number of steps, the indifference point is calculated (a mean of the two last certain and immediate alternatives). The indifference point is a subjective equivalent of the delayed lottery. The set of indifference points assigned to each postponement periods forms a discounting curve—separate for each researched lottery (Figure 1).

In the next step, the general discounting strength is calculated as “area under the curve” (Myerson et al., 2001). The subjective value (measured on vertical axis) is scaled to the starting value (e.g., if the 0.9 probability of gaining $5.000 in 1 week is equal to $4.850, then the subjective equivalent is scaled in comparison with maximal possible value, thus: 4.850/5.000 = 0.98). The researched time range (7–730 days, measured on horizontal axis) is also scaled on 0–1 scale. The area under the curve is then calculated: the scaled equivalents are connected with lines and the cumulated area of trapeze *AUC*_1_ (*t*_1_, *t*_2_, *e*_2_, *e*_1_) and *AUC*_2_ (*t*_2_, *t*_3_, *e*_2_, *e*_3_) (as calculated by Equations 1 and 2) is added:

(1) $$AUC_{1}=\frac{\left(e_{1}+{\text{e}}_{2}\right)*\left(t_{2}-t_{1}\right)}{2}$$

(2) $$AUC_{2}=\frac{\left(e_{2}+{\text{e}}_{3}\right)*\left(t_{3}-t_{2}\right)}{2}$$

The smaller the area under the curve, the faster is the loss of value in the process of discounting. Usually, the procedure of establishing the indifference point requires six steps, comprising 54 choices when discounting nine delayed lotteries. Having in mind that all choices/screens are similar—both in content and graphic—and one of the alternatives is constant, the procedure is potentially tedious. Moreover, the reported face validity of the method seems to be low (Odum, 2011a). Moreover, the studies using adjusting method provide a high ratio of irrational choices (excluding up to 20–50% of participants, (see Appelt et al., 2011; Bialek and Sawicki, 2014) and low time consistency of measured preferences, which results in noisy data and sometimes even brings contradictory conclusions for researchers (for a broad discussion see Frederick et al., 2002).

#### Conjoint analysis

Conjoint analysis, although rooted in the conjoint measurement theory (Luce and Tukey, 1964; Krantz et al., 1971) has been mostly used in the marketing (Green and Srinivasan, 1990) and applied psychology fields (Green and Srinivasan, 1990; Brocke et al., 2004; Takemura et al., 2006; Caruso et al., 2009; Czupryna et al., in press; Takemura, 2014).

The main idea of conjoint analysis is based on making trade-off by the decision makers. To make the trade-off possible the alternative needs to be described by at least two characteristics—specifically for delayed lotteries: postponement and probability; each has to be defined on minimum two levels. The number of choice sets as well as the number of lotteries (usually 3–5) forming the particular choice set is a subjective decision of the researcher (Orme, 2010). Because presenting all possible combination of all profiles would be to exhausting for individuals (one can draw 84 different sets of three profiles out of nine possible) they are presented with only a part of choice sets: the conjoint software creates multiple version of study for respondents to get balanced (each level of attribute appearing the same number of times) and orthogonal design (each level of attribute appearing together with other attribute similar number of times (Kuhfeld et al., 1994; Sawtooth Software, 2013a). This means, that every participant was presented with different version of the conjoint task. Contrary to adjusting the consecutive choices made by an individual do not influence options presented further in the task.

The decision maker is choosing one best profile among the set of profiles presented at particular step. In our case profiles were nine alternatives with unique combination of probability and delay, and which were displayed in sets of three. First step of analysis in choice based conjoint is calculating the “win ratio” of each profile, this proportion it was chosen to times it was presented. Then the regression analysis conducted, in which the win ratio is a dependent variable and the dummy coded attribute levels and its 2nd level interactions serve as predictors. The regressions produces only group level beta scores, that are treated as “utilities” of each specific attribute level, and to derive the utilities on the individual level a Hierarchical Bayes method is used (Allenby et al., 1995; Lenk et al., 1996). This method estimates individual preference (*b*), populations preference (*a*), and a variance/covariance matrix (*D*) by improving the starting model in which *b* is assumed to be randomly drawn from a multivariate normal distribution:

b~N(a,D)

This starting model has an initial estimates of *a, b*, and *D*. The estimate of *b* is the “win ratio” of *a* specific attribute level; *a* has all elements equal to zero, and for *D* the initial variance is an unity and covariance zero (Johnson, 2000). Then an algorithm repeats following three steps (Sawtooth Software, 2009) in user specified number iterations (in our study 20.000):

1. Given current estimates of the *b* and *D*, estimate the vector *a* of means of the distribution.
2. Given current estimates of the *b*, and *a*, estimate the matrix *D* of variance/covariance.
3. Given current estimates of *a*, and *D*, estimate a new *b* vector for each respondent.

After each iteration the individual preference (*b*) general preference (*a*) and variance/covariance matrix (*D*) estimations are updated. The detailed discussion of statistical methods is beyond the scope of this paper but more details can be found in the literature (Johnson, 2000; Green et al., 2001; Bradlow, 2005; Sawtooth Software, 2009, 2013b; Chib, 2011).

Because the utility of a profile is a sum of utilities of its components (e.g., utility of 0.1 chance receiving a reward in 2 years is a sum of utility of 0.1 probability and utility of 24 months delay) we calculated for each researched probability level (e.g., 0.1) how its utility decreases in time (as described by its utility in three time points: 1 week, 3 months, 2 years), see Figure 1. The aggregate area of the trapezes formed under the curve can be interpreted as time discounting strength of particular lottery (with smaller area under the curve interpreted as faster loss of value in the process of discounting).

In case of single respondent answers being inconsistent, the Bayesian regression estimation influences the individual utility of attributes by the population mean (high “Bayesian Shrinkage,” see Lenk et al., 1996; Orme and Howell, 2009). That could pose limitation for between-subjects comparison, as on individual level the less congruent respondents are “altered” toward populations' average utility. Moreover, the hierarchical Bayes procedure is iterative, thus, depending on the random seed you use, you will achieve slightly different part worth's from subsequent runs. However, the differences should converge toward zero as the number of iterations increases (Johnson, 2000). Still however it limits the between-study comparisons.

### Study 1

Before the start of the study, participants provided written informed consent. The Kozminski University Ethics Committee approved the consent form as well as the study procedure. In a 2-stage study, the group of participants made a series of choices using the adjusting procedure; 3 weeks later, the same group was tested with the conjoint procedure. On both occasions participants also selected one preferred lottery from three pairs presented (three choice tasks). Participants made their choices in a hypothetical situation with no real monetary compensation. Bickel and collaborates (Johnson and Bickel, 2002; Bickel et al., 2009) found that hypothetical financial intertemporal decisions is similar to those with real money while other researchers (Chabris et al., 2008; Reimers et al., 2009) stated that hypothetical tasks predict real-life choices well.

#### Participants

A group of polish speaking Kozminski University management students (*n* = 27, 70% females; aged *M* = 21.25, *SD* = 1.47) was recruited from a “Psychology of personality and individual differences” course. Participants were informed about the goals of the study and debriefed after the second part of the study. They received course credit for their participation. All participants performed two parts of the study (Choice Task + Adjusting, and 3 weeks later Choice Task and Conjoint).

#### Procedure

**Figure 2:**
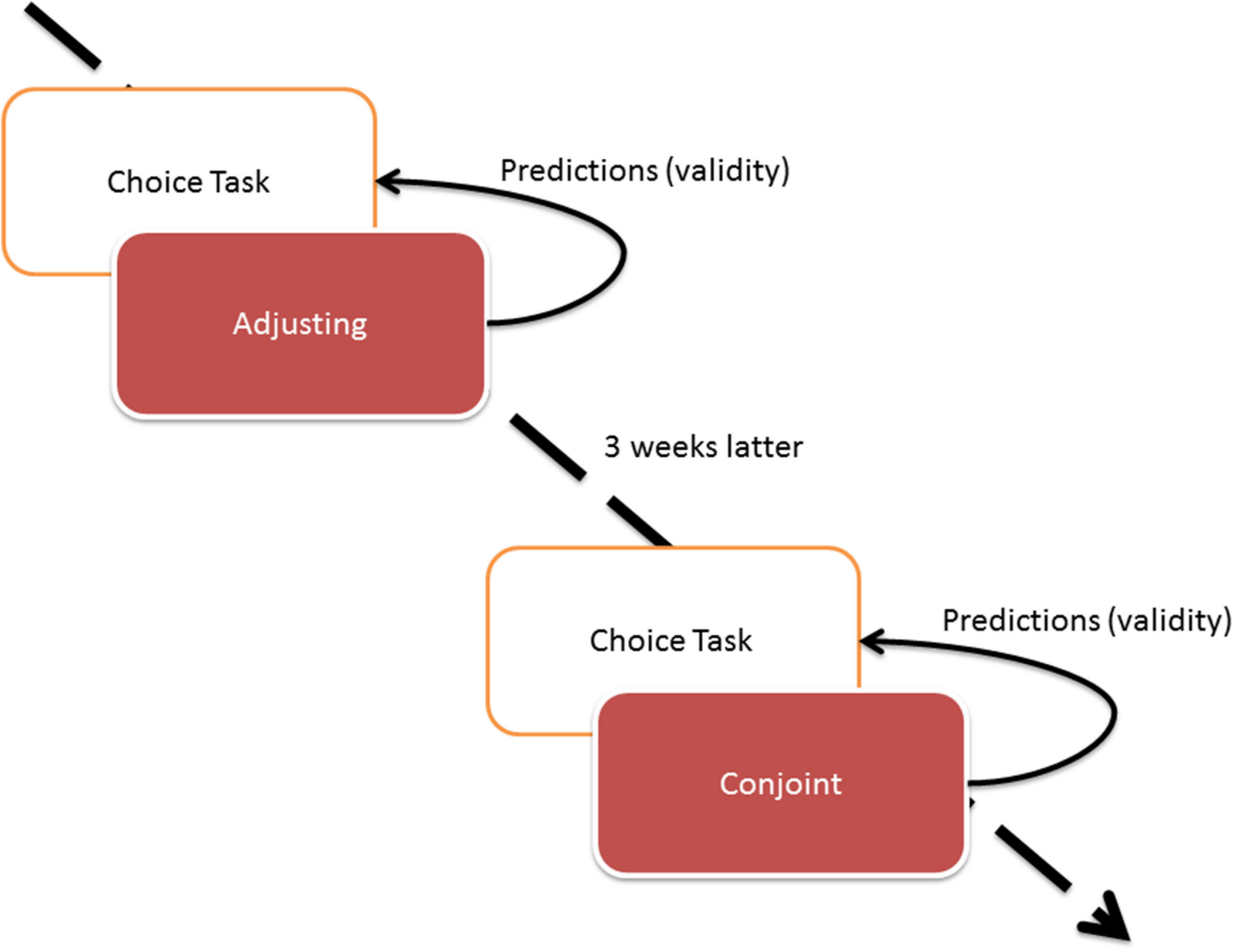
**The Study 1 design**.

The design of the study is presented in Figure 2.

The study consisted of two waves. The first wave comprised choice tasks and adjusting while the second one involved choice and conjoint tasks. Because choice task was designed as an external validity check (dependent variable for regression), we did not want its results to be influenced by choices made in conjoint or adjusting conditions (independent variable), thus we designed the study where choice task was completed first in both waves of the study.

All assessed delayed lotteries had the same value of 1.000 PLN (approximately $300, equal to approximately 70% of minimal net monthly salary in Poland). In both the conjoint and adjusting tasks, the individuals assessed nine delayed lotteries defined by three delay periods (1 week, 3 months, and 2 years), and three possible probabilities (0.01, 0.1, and 0.7). The probability and delay span were chosen arbitrary to cover a wide range of lotteries—from certain and immediate (0.7 in a week's time) to hardly probable and postponed (0.01 in 2 years). Of course, data that are more reliable could be obtained if more levels of delay and probability would have been used. The reason we decided to limit the levels to three was not to produce too long and exhausting study, as adjusting would have to consist of 96 sets of choices for 4 × 4 study design.

***Adjusting***. The nine lotteries were each assessed in six steps to establish their monetary indifference points (9 × 6 = 54 dichotomous choices for each participant). After collecting nine equivalents, the area under the curve for three lotteries (0.01; 0.1; 0.7) has been calculated for every participant (Table 1). In the end, each participant's discounting strength was described with three numbers, representing three areas under the curve. Bigger area under the curve indicates smaller discounting strength of particular delayed lottery.

**Table 1:** **Choice Tasks in study 1**.

**Choice task**	**Lottery A**			**Lottery B**		**Ratio the lottery A was chosen, in %**	
	**Probability**	**Delay**		**Probability**	**Delay**	**Wave 1**	**Wave 2**
Decision 1	0.01	1 week	or	0.1	3 months	30	22^**^
Decision 2	0.01	3 months	or	0.1	2 years	67	67^**^
Decision 3	0.1	3 months	or	0.7	2 years	59	48^*^

Kappa correlation was significant at

* p < 0.05;

** p < 0.01.

***Conjoint***. Participants assessed the same nine lotteries as in the adjusting wave, however, with a different task configuration. In the subsequent 25 conjoint tasks^1^, the participants chose one best lottery from three presented to them on the screen; thus, conjoint study participants were asked to make half as many choices as in the adjusting part; however, the choices were more complex, forcing participants to make trade-off between time and probability (while adjusting trades off the monetary value and postponement separately for each given probability). The conjoint analysis produced utilities for each attribute level, allowing us to calculate the cumulative utility for each of the nine lotteries. A procedure of drawing curves for each probability of gain was done similar to the one for adjusting. As a result, three values describing a discounting strength of three lotteries (0.01; 0.1; 0.7) for each participant were calculated.

To conduct the study and compute the utilities, we used Sawtooth Software; however, the same values can be conducted using other software—both commercial SPSS (see procedure explained in Walesiak and Bąk, 2000) and non-commercial R package with available online scripts (Bąk and Bartłomowicz, 2012; Bąk, 2013).

***Choice Task (CT)***. To evaluate the predictive ability of both adjusting and conjoint an external task was required. We have designed a Choice Task, in which participants were presented with several dichotomous choices of delayed lotteries, as presented in Table 1. Each time, they had to declare which one of the two they prefer. All Choice Task consisted lotteries were also evaluated in other tasks. These tasks included also other lotteries and differed for participant: in conjoint one had to choose one out of three alternatives while in adjusting one had to provide certain and immediate equivalent for each of the lotteries.

## Results

### Study 1 results

Discounting equivalents and cumulative utilities calculated for each lottery with both adjusting and conjoint methods are presented in Table 2.

**Table 2:** **Discounting equivalents (cumulative utilities) calculated for each lottery with adjusting (conjoint) method within Study 1**.

**Lottery**	**Adjusting (*n* = 27)**		**Conjoint (*n* = 27)**	
	**Mean equivalent**	***SD***	**Cumulative utility**	***SD***
0.01 chance in 1 week	122.741	108.439	−2.896	3.263
0.1 chance in 1 week	218.852	215.483	3.572	1.549
0.7 chance in 1 week	518.593	224.376	11.339	2.226
0.01 chance in 3 months	134.296	118.571	−5.361	2.320
0.1 chance in 3 months	252.444	234.225	1.107	1.070
0.7 chance in 3 months	480.519	245.952	8.874	2.373
0.01 chance in 2 years	96.111	105.240	−12.446	2.334
0.1 chance in 2 years	179.407	216.335	−5.978	2.331
0.7 chance in 2 years	343.889	302.208	1.789	3.400

As the Choice Task was repeatedly presented to individuals, the time stability of preferences was tested. It showed to be considerably high (Kappa correlation for decision 1, 2, and 3 accounts to 0.617^**^; 0.667^**^; 0.338^*^ respectively, all significant with *p* < 0.05), giving evidence of moderate time stability of the preference for delayed and uncertain pay-offs. This is consistent with the literature suggesting delay discounting as a trait variable (Odum, 2011a,b); thus, we expected the discounting measurement methods also to provide stable and repeatable parameters.

#### External validity of conjoint and adjusting

**Figure 3:**
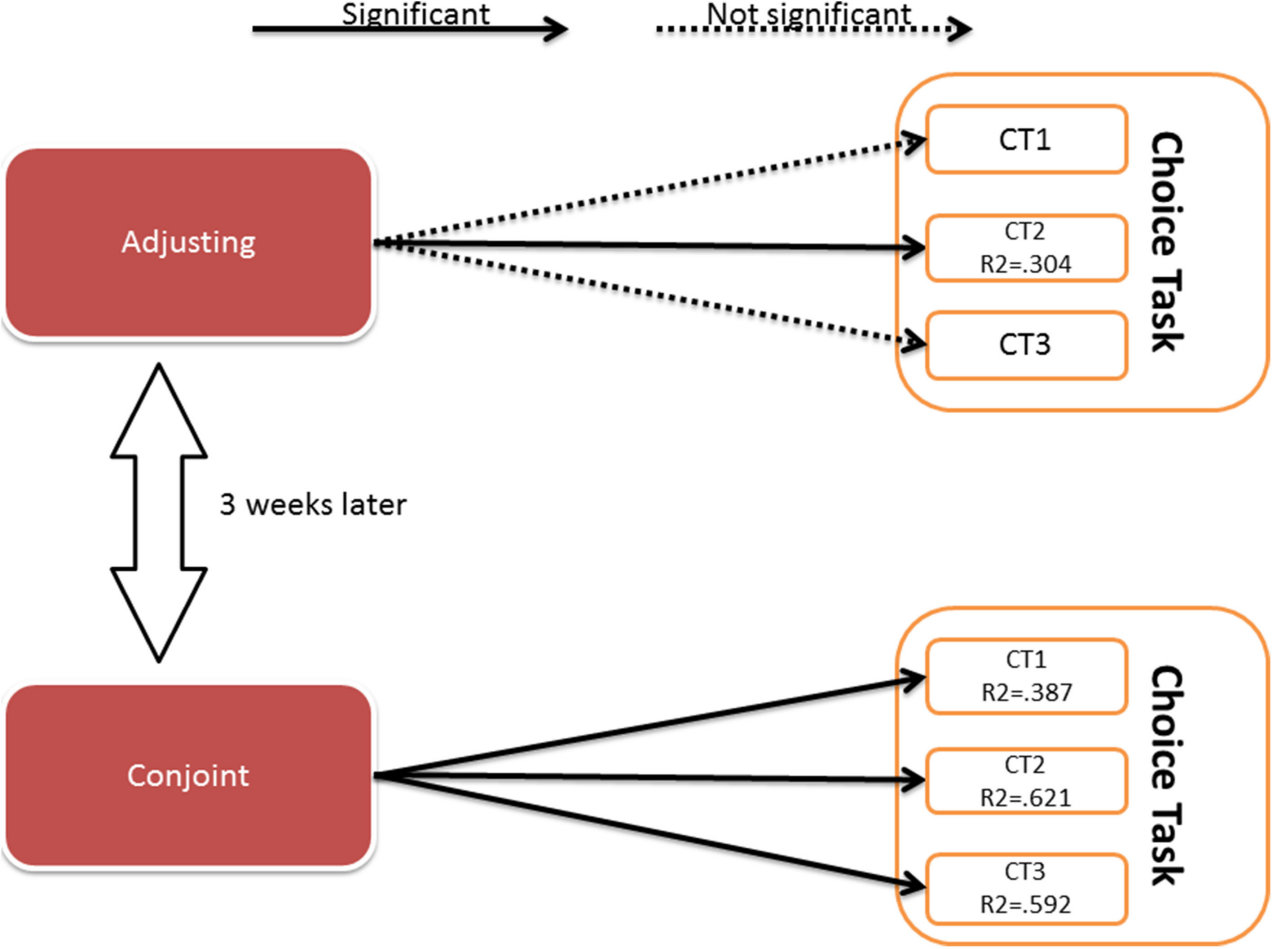
**Results of the binary logistic regression**. Details of the analysis are presented in the Appendix 1 (Supplementary Material).

Next, we analyzed the discounting strength of each individual. These parameters (from both conjoint and adjusting) served as predictors in a binary logistic regression with conditional backwards method. The binary decision in the choice task was the dependent, predicted value. The results of the three analyses (for three decisions in choice task) are presented in Figure 3.

The details of the analysis are presented in Appendix 1 (Supplementary Material).

The results showed that the discounting strength parameters obtained by the conjoint method predicted all the decisions in the choice task, although the parameters obtained by adjusting predicted only one decision. The predictive value of the created models was of moderate strength (Nagerkelke pseudo *R*^2^ ranges between 0.3 and 0.6). The Akaike Information Criterion (AIC) has lower values for conjoint model indicating that this model fits the data relatively better than adjusting model.

#### Study 1 discussion

Our primary findings suggest higher predictive accuracy of conjoint compared to adjusting method. Discounting parameters calculated using conjoint predicted all choices on the choice task, but only one choice (decision 2) was predicted by the parameters calculated using adjusting method. A relatively small number of tested individuals and moderate variance of the choice task explained by the binary regression models are limitations of the Study 1. Also the use of conjoint method after participants have done the adjusting could increase the self-awareness of individuals and thus increase the consistency of answers in the second wave of the study. To avoid of these flaws and to have insight also on the time stability of the preferences estimated with both methods, we conducted Study 2 with improved design.

## Methods – study 2

### Participants

Sixty participants (67% females; M age = 27.25, *SD* = 5.36) attending the class “Psychology of consumer behavior” took part in the first study session (T_0_). Twenty-two of them took part in the repeated measurement (T_1_) 3 weeks later (68% females; mean age = 25.59 *SD* = 4.08). Participants were informed about the goals of the study and debriefed after the second wave of the study.

### Procedure

**Figure 4:**
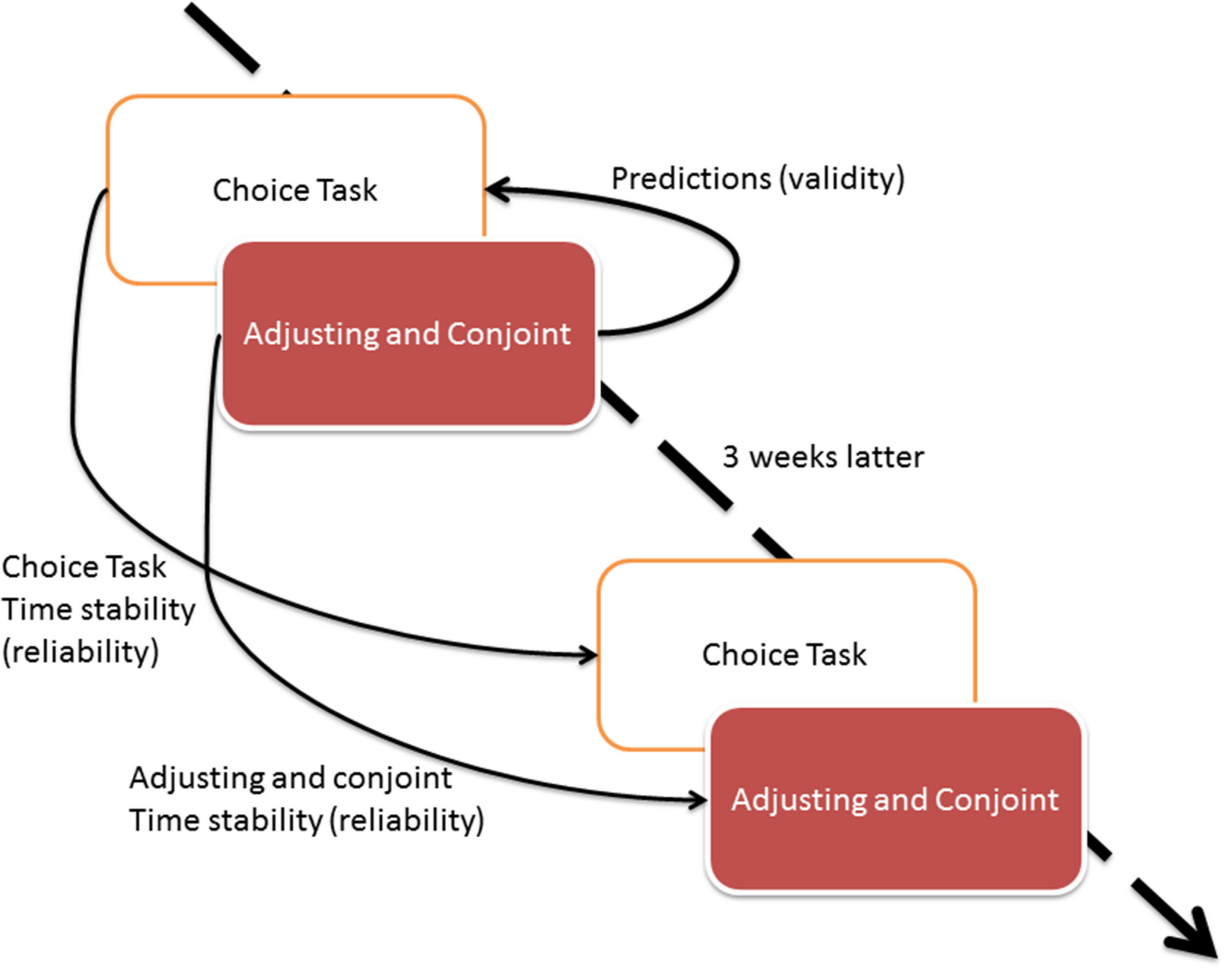
**The Study 2 design**.

The design of Study 2 is presented in Figure 4.

First, all individuals completed the same choice task as in Study 1. Next, they assessed nine delayed lotteries with both adjusting and conjoint methods. The lotteries consisted of identical levels of attributes—probability and delay—as in Study 1. Conjoint and adjusting methods were presented to participants in random order; thus, some participants solved conjoint task first while others completed adjusting task first. The procedure was repeated in a follow-up study 3 weeks later. The validity assessment was conducted only for the first wave of study to avoid the effect of recall of the decisions made 3 weeks later.

The design of Study 2 allowed us to assess the time stability of all methods measuring the preferences in delayed lotteries: choice task, conjoint and adjusting methods. Because in Study 2 all tasks have been done the same day, we were able to conduct single regression analysis with all the parameters from conjoint and adjusting instead of conducting separate analysis for adjusting and conjoint methods.

## Study 2 results

### Choice task

Moderate consistency of the choice task over 3 weeks was observed. Kappa correlation parameter for task 1 was not significant (none of 1st wave lottery choosers repeated their choice in follow-up study) while the correlation of other choices was 0.431, *p* < 0.05 and 0.486, *p* < 0.01 for task 2 and 3, respectively. The exact ratio of choices made by individuals is presented in Table 3.

**Table 3:** **Results of the choice tasks in Study 2**.

**Choice task**	**Lottery A**			**Lottery B**		**Ratio the lottery A was chosen, in %**	
	**Probability**	**Delay**		**Probability**	**Delay**	**Wave 1, *N* = 60**	**Wave 2, *N* = 22**
Decision 1	0.01	1 week	or	0.1	3 months	8	4
Decision 2	0.01	3 months	or	0.1	2 years	53	50^**^
Decision 3	0.1	3 months	or	0.7	2 years	37	27^**^

Kappa correlation was significant at

** p < 0.01.

### External validity of conjoint and adjusting

Discounting equivalents and cumulative utilities calculated for each lottery with adjusting and conjoint methods are presented in Table 4.

**Table 4:** **Discounting equivalents (cumulative utilities) calculated for each lottery with adjusting (conjoint) method within Study 2**.

**Lottery**	**Adjusting (*n* = 60)**		**Conjoint (*n* = 60)**	
	**Mean equivalent**	***SD***	**Cumulative utility**	***SD***
0.01 chance in 1 week	162.933	317.136	−6.609	3.819
0.1 chance in 1 week	157.800	259.813	2.464	1.200
0.7 chance in 1 week	421.933	295.145	15.343	4.395
0.01 chance in 3 months	96.317	191.769	−8.279	2.946
0.1 chance in 3 months	103.100	157.462	−0.003	1.416
0.7 chance in 3 months	425.567	285.879	11.044	2.836
0.01 chance in 2 years	94.683	215.654	−13.319	3.318
0.1 chance in 2 years	141.100	244.955	−5.738	1.725
0.7 chance in 2 years	256.300	276.213	5.096	5.050

**Figure 5:**
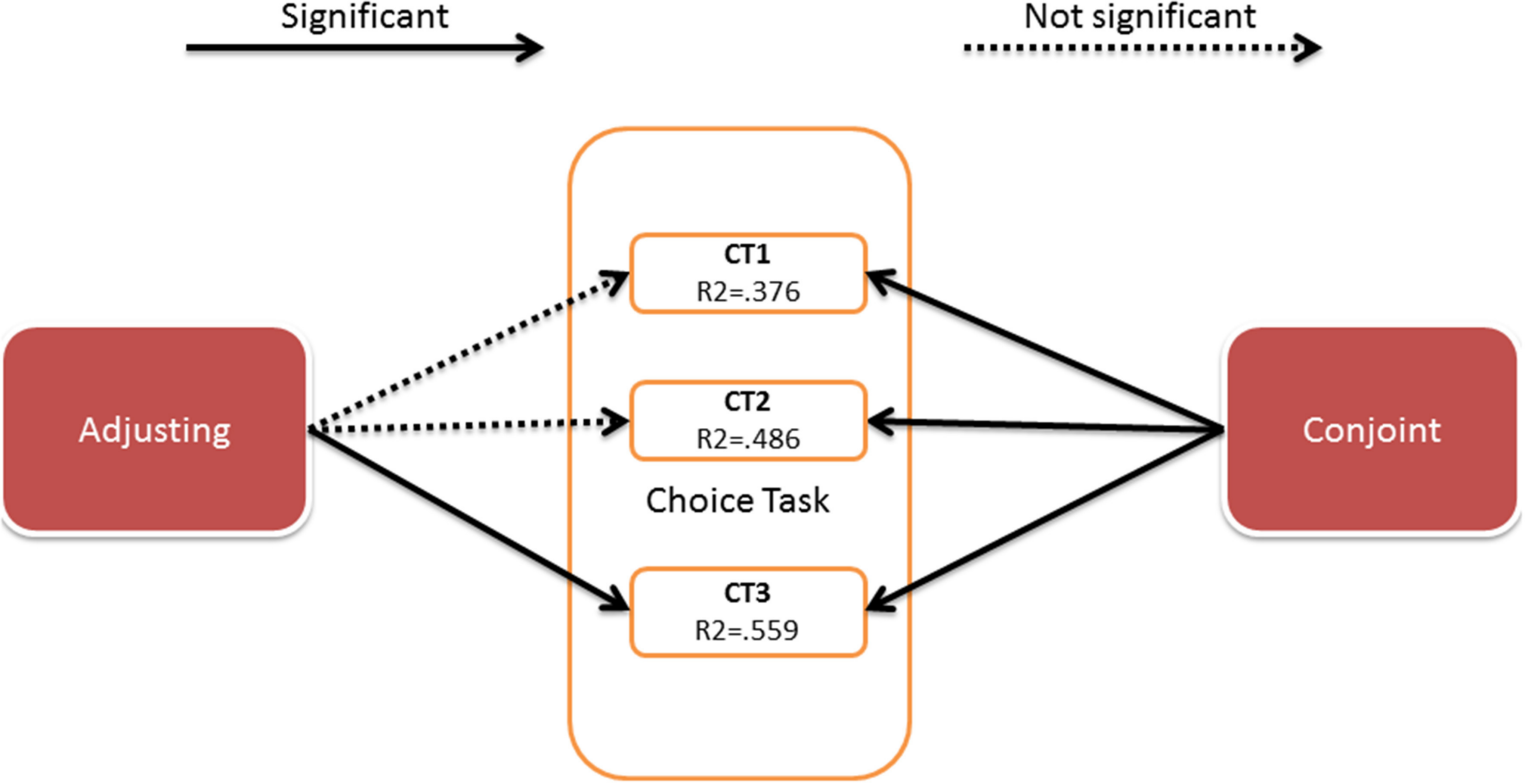
**Results of the binary logistic regression**. Details of the analysis are presented in the Appendix 2 (Supplementary Material).

We conducted a validity check using logistic regression. We expected the area under the curve parameter to predict the decisions made in the Choice Task the same day. The analysis of data from all 60 individuals was conducted; the binary logistic regression with conditional backward method was used and the results are shown in Figure 5.

The details of the analysis are presented in Appendix 2 (Supplementary Material).

As in Study 1, discounting parameters obtained by the conjoint method predicted all choices made on the choice task, and only one choice was predicted by the adjusting method. The predictive value of the created models is of moderate strength (pseudo *R*^2^ range 0.4–0.7).

### Time consistency of the measurement method

We tested time consistency of the measurement method by correlating the corresponding discounting parameters obtained over 3 weeks (Table 5). This means that we correlated the time discounting area under the curve for each lottery (0.01; 0.1; 0.7) with the same discounting strength of the same individual obtained in follow up measurement.

**Table 5:** **Time stability of discounting parameter (area under the curve) over 3 weeks, *N* = 22**.

	**Conjoint**	**Adjusting**
0.01 lottery discounted 1 week, 3 months, 2 years	0.585^**^	−0.131
0.10 lottery discounted 1 week, 3 months, 2 years	0.520^*^	−0.131
0.70 lottery discounted 1 week, 3 months, 2 years	0.577^**^	0.321

Significant at

* p < 0.05;

** p < 0.01

No correlations of discounting parameters were observed between the two measurements of the adjusting method while the conjoint method revealed moderate correlation between subsequent measurements^2^. The above-mentioned results confirm the time stability of the individual discounting strength, and provide an additional argument to define discounting strength as a stable personal trait.

## General discussion

Conjoint, with its higher validity and time stability, can be considered as a better tool to describe ones preferences in delayed lotteries. The delayed lotteries are a combination of one's impulsivity (when considering time discounting) and his or hers probability weighting functions (when considering risk). The probability weighting function is an essential parameter form prospect theory (Kahneman and Tversky, 1979), it describes ones tendency to overestimate low probabilities and underestimate high probabilities. Considering the values of probability weighting functions we are able to broadly predict a specific choices made by an individual when deciding between several alternatives including risk. Recent researches focuses on the interaction of both delay and risk in order to find a suitable model that fits the collected data (Vanderveldt et al., 2015; Weatherly et al., in press). Our reported here data can be helpful in attempts this question, as we propose a more reliable method of collecting the data, meanly by conjoint, and thanks to that potentially increase the chance to understand the interactive nature of delay and risk discounting.

We are unable to say, if conjoint is more efficient in calculating the probability weighting functions or time discount, or the interaction of both, but we will discuss the essential differences between conjoint and adjusting in the area of delayed lotteries and propose three explanations why the first method seems to be better than the second one.

### Why the adjusting method does not work well for measuring delayed lotteries?

The domain of delayed lotteries is representative of real life situations (e.g., going on camping trip now even though the weather is not so great or wait a week, waiting for a potentially better weather) but has quite a short experimental tradition. The goal of the paper was to introduce a relatively new research method, the conjoint analysis, and compare it with the most prominent one, adjusting analysis. This study has an exploratory character, and it should not be treated as an evidence of one method being superior over other. We suggest that researchers consider both methods in their studies to triangulate their findings.

At the general level, the preference in delayed lotteries was considerably stable over time, which means that people answered in the same or similar way on the choice task. Some claim that the time discounting preference is a stable psychological trait (Odum, 2011a,b).

The conjoint method showed greater predictive accuracy for real-life choices compared to the adjusting method. We were able to predict decisions made in the choice task using discounting parameters computed based on the conjoint rather than adjusting method.

The time consistency of adjusting method was lower than that of conjoint method: specifically, the results of one measurement of adjusting did not correlate with follow up measurement while other methods used in our studies (Choice Task and conjoint) correlated significantly. This provides evidence that discounting strength does not change much over time, thus the problem lays rather in adjusting method itself.

We offer three different explanations for why the adjusting method does not work well for measuring delayed lotteries, although it provides valid and reliable data in other areas of research:

#### Abstractness of the data

Potentially adjusting method is not optimal for measuring the preferences in delayed lotteries due to the abstractness of information communicated to participants, as lay people are not particularly good at understanding and evaluating probabilities (Hoffrage et al., 2000; Siegrist et al., 2008; Tyszka and Sawicki, 2011). Adjusting works well when it refers to simple perceptual stimuli, which do not involve higher level cognition (as in psychophysics—see Gescheider, 1997). In the studies presented in this article, adjusting method was used to determine the equivalents for delayed lotteries, which do trigger higher level of cognition (specifically when evaluating the probability). Because of the difference in abstractness of data, adjusting can work less efficient in delayed lotteries compared to other fields of research.

#### Motivation

Because in adjusting, one alternative from several consecutive choices is constant, one can interpret the specific situation as a chance to “sell” the delayed lottery at highest price possible; thus, one can alter own decisions to achieve the goal (maximize immediate gain) instead to find a point of indifference between two alternatives. After first few choices, individual can realize that after choosing immediate offer, the next immediate offer decreases and he or she “loses.” Thus, in order to get the maximum, one should reject first few offers (certain and immediate alternative), as she/he is convinced that he/she will soon receive more attractive offers. This hypothetically influences the value of equivalents in adjusting task.

This problem do not appear in psychophysics, where adjusting is also used. In this type of tasks, an individual has to adjust stimuli, like light or weight, as these modalities have no positive or negative valence (unlike money). Thus, no motivation to maximize the “gain” occurs and individual are more likely to follow the instructions and match two stimuli.

The alternative motivation problem also does not apply to conjoint method in which individual chooses always the most attractive offer from several alternatives. An individual has no feeling that presented later alternatives deteriorated because of her/his previous choices.

#### Sensitivity to change

People are sensitive especially to changes in values and not the values itself (Kahneman and Tversky, 1979); thus, parameter that changes could increase respondents' attention more than would a parameter that does not change. Adjusting design includes one constant alternative and one that is adjusting while conjoint design presents individuals with sets of new alternatives in each step. This can encourage individuals to pay greater attention to conjoint rather than adjusting tasks.

While evaluating the delayed lotteries, some people use simple heuristics and try to simplify the decision process, similarly to the editing stage of the prospect theory (Kahneman and Tversky, 1979). Instead of compensating for delay, they can use more simple decision rules, such as lexicographical (Tyszka, 2010), thus concentrate only on dominant attribute, and took other attribute into consideration only if set choice is not differentiated by dominant attribute. Participants can use this strategy when completing conjoint but not adjusting tasks since in adjusting task choices consist always of two alternatives different on all attribute levels. Assuming that the lexicographical strategy (compared to compensational strategy) is easier to apply in a decision process and the participants cannot use it in adjusting task, their performance in this task can be less efficient.

### Limitations and further research

Our study has several significant limitations. The relatively small sample size is a possible limitation of our study. Moreover, one could expect stronger effect when offering real monetary incentives instead of fictitious ones.

The conjoint and adjusting study design could be improved in further studies. First, assessing more than three probabilities and postponements could provide more precise estimates of discounting strength, as measured by area under the curve. However, such a precision could only be achieved by the cost of: respondents increased fatigue (adjusting task) or the increase of required sample size (conjoint task).

Our arbitrary decision to use specific probability range (1–70%) and reward postponement (1 week–2 years) can be questioned, because one can discuss that a greater span of both attributes could significantly increase the subjective importance of each. Moreover, the middle point of these spans can be selected differently, e.g., the probability of 0.35 instead of 0.10 and delay of 1 year instead of 3 months. This change would allow more precise calculation of the area under the curve.

In this study, the choice task included choices created using the same levels of attributes as those used in conjoint and adjusting tasks. We believe that our study would be even more interesting if the choice task consisted of different probabilities and postponements compared to those used in adjusting in conjoint tasks. The ability to predict the choices external to experimental task would be an important feature of any research method.

We believe that adjusting method is simply too complicated for a respondent because it requires him/her to pay extreme attention to sets of merely distinguishable choices. Because the adjusting method—designed as a multi-branched choice tree—presents new choices that depend on respondents previous choices, the concentration gaps increase a chance of individuals making single irrational choice, influencing the whole set of choices as well as the final equivalent. The software usually allows respondent to move back to previous choice to correct it, but in practice, individuals hardly use this option. Thus, researchers should look for new methods to be used in probability discounting studies. The new methods should be resistant to single irrational response made by participant possibly by averaging several choices.

To conclude, in light of the data presented, conjoint should be considered as an alternative discounting measurement method. The authors hope that this study's findings will help scientists interested in the topic of discounting choose the optimal method. We also recommend using the conjoint method as an additional tool in triangulation procedures to validate the results of the adjusting method and designing new studies with the use of both methods. Getting similar results with both methods would increase the validity of conclusions, moving the area of research forward.

### Conflict of interest statement

The authors declare that the research was conducted in the absence of any commercial or financial relationships that could be construed as a potential conflict of interest.

## Footnotes

^1^The optimal number of sets of choices has been defined as a rule of a thumb into 25 (Orme, 2010).

^2^Although we do not report these results here, the discounting strength (area under the curve) measured with adjusting method does not correlate with the same parameter measured with conjoint method.
