# Supplementary material for: Introducing conjoint analysis method into delayed lotteries studies: its validity and time stability are higher than in adjusting
Source: Front Psychol. 2015 Jan 28;6:23. doi: 10.3389/fpsyg.2015.00023 (PMC4309168; doi:10.3389/fpsyg.2015.00023)
Supplement: Supplementary file 1 [file DataSheet1.DOCX]

1. **Supplementary Material**

Appendix 1.

Table 6. Results of binary logistic regression with conditional backward method in Study 1

|  |  | Decision 1* | | Decision 2* | | Decision 3* | |
| --- | --- | --- | --- | --- | --- | --- | --- |
| Dependent variable | Predictor | B | Odds | B | Odds | B | Odds |
| Decision made in the wave 1 | adjusting .01 | - | - | - | - | - | - |
|  | adjusting .1 | - | - | -.005 | .993 | - | - |
|  | adjusting .7 | - | - | .007 | 1.005 | - | - |
|  | constant | .865 | 2.375 | -2.528 | .08 | -.375 | .688 |
| Chi-square | | - | | 6.675; df=2;p<.05 | | - | |
| Nagerkelke pseudo R-square | | - | | .304 | | - | |
| concordance rate | | .50 | | .784 | | .50 | |
| Akaike Information Criterion (AIC) | | 34.815 | | 33.696 | | 38.499 | |
| Decision made in the wave 2 | conjoint .01 | - | - | 1.772 | 5.882 | - | - |
|  | conjoint .1 | - | - | - | - | 1.375 | 3.995 |
|  | conjoint .7 | .646 | 1.908 | 2.249 | 9.482 | .683 | 1.980 |
|  | constant | -14.858 | .001 | -80.059 | .001 | -42.412 | .001 |
| Chi-square | | 7.858; df=1; p<.01 | | 15.997, df=2; p<.001 | | 15.830; df=2; p<.001 | |
| Nagerkelke pseudo R-square | | .387 | | .621 | | .592 | |
| concordance rate | | .833 | | .920 | | .896 | |
| Akaike Information Criterion (AIC) | | 24.746 | | 24.374 | | 27.562 | |

* The dependent value was a choice made in the Choice Task

Appendix 2.

Table 7. Results of binary logistic regression with conditional backward method in Study 2

|  | Decision 1* | | Decision 2* | | Decision 3* | |
| --- | --- | --- | --- | --- | --- | --- |
| Predictor | B | Odds | B | Odds | B | Odds |
| adjusting .01 | - | - | - | - | - | - |
| adjusting .1 | - | - | - | - | -.005 | .995 |
| adjusting .7 | - | - | - | - | - | - |
| conjoint .01 | - | - | - | - | -1.140 | .320 |
| conjoint .1 | - | - | - | - | 1.385 | 3.996 |
| conjoint .7 | .570 | 1.768 | .516 | 1.676 | - | - |
| constant | -12.447 | .000 | -15.203 | .000 | -12.230 | .000 |
| Chi-square | 10.761; df=1; p<.001 | | 27.125; df=1; p<.0001 | | 31.522; df=3; p<.0001 | |
| Nagerkelke pseudo R-square | .376 | | .486 | | .559 | |
| concordance rate | .891 | | .872 | | .879 | |
| Akaike Information Criterion (AIC) | 27.659 | | 59.786 | | 55.337 | |

* The dependent value was a choice made in the Choice Task
